# Inhibition of Activity of GABA Transporter GAT1 by **δ**-Opioid Receptor

**DOI:** 10.1155/2012/818451

**Published:** 2012-12-25

**Authors:** Lu Pu, Nanjie Xu, Peng Xia, Quanbao Gu, Shuanglai Ren, Thomas Fucke, Gang Pei, Wolfgang Schwarz

**Affiliations:** ^1^Max-Planck-Institute for Biophysics, Max-von-Laue-Straße 3, 60438 Frankfurt, Germany; ^2^Laboratory of Molecular Cell Biology, Institute of Biochemistry and Cell Biology and Max-Planck Guest Laboratory, Shanghai Institutes for Biological Sciences, Chinese Academy of Sciences, 320 Yue Yang Road, Shanghai 200031, China; ^3^Department of Neurobiology and Behavior, Center for the Neurobiology of Learning and Memory, University of California, Irvine, CA 92697, USA; ^4^Shanghai Research Center for Acupuncture and Meridians, 199 Guoshoujing Road, Shanghai 201203, China; ^5^NingXia Key Lab of Cerebrocranial Disease, Ningxia Medical University, 1160 Shengli Street, Ningxia Hui Autonomous Region, Yinchuan 750004, China; ^6^Central Institute of Mental Health, BCCN Heidelberg-Mannheim, J5, 68159 Mannheim, Germany; ^7^Institute for Biophysics, Goethe-University Frankfurt, Max-von-Laue-Straße 1, 60438 Frankfurt, Germany

## Abstract

Analgesia is a well-documented effect of acupuncture. A critical role in pain sensation plays the nervous system, including the GABAergic system and opioid receptor (OR) activation. Here we investigated regulation of GABA transporter GAT1 by *δ*OR in rats and in *Xenopus* oocytes. Synaptosomes of brain from rats chronically exposed to opiates exhibited reduced GABA uptake, indicating that GABA transport might be regulated by opioid receptors. For further investigation we have expressed GAT1 of mouse brain together with mouse *δ*OR and *μ*OR in *Xenopus* oocytes. The function of GAT1 was analyzed in terms of Na^+^-dependent [^3^H]GABA uptake as well as GAT1-mediated currents. Coexpression of *δ*OR led to reduced number of fully functional GAT1 transporters, reduced substrate translocation, and GAT1-mediated current. Activation of *δ*OR further reduced the rate of GABA uptake as well as GAT1-mediated current. Coexpression of *μ*OR, as well as *μ*OR activation, affected neither the number of transporters, nor rate of GABA uptake, nor GAT1-mediated current. Inhibition of GAT1-mediated current by activation of *δ*OR was confirmed in whole-cell patch-clamp experiments on rat brain slices of periaqueductal gray. We conclude that inhibition of GAT1 function will strengthen the inhibitory action of the GABAergic system and hence may contribute to acupuncture-induced analgesia.

## 1. Introduction

Neurotransmitter transporters play a key role in the regulation of synaptic transmission. Glutamate and GABA are the dominating excitatory and inhibitory neurotransmitters in the mammalian brain, respectively. The predominate transporters controlling glutamate and GABA in the CNS are the excitatory neurotransmitter transporter EAAC1 [1–3] and the GABA transporter GAT1 [4].

It is generally accepted that pain sensation can be suppressed by acupuncture and that regulation of the glutamatergic and the GABAergic systems is involved in pain sensation [5]. Inhibition of the excitatory glutamatergic system and stimulation of the inhibitory system will contribute to analgesia. It could be demonstrated that inhibition of excitatory amino acid (EA) receptors [6, 7] and stimulation of GABA-A and GABA-B receptor [5, 8] resulted in pain suppression. Indirect reduction of EA-receptor activity may also be achieved by reduced glutamate concentration in the synaptic cleft, and reduction of glutamate concentration can be achieved by stimulating EAAC activity [9, 10]. In analogy to stimulation of EAAC1, we may expect for the GABAergic system that inhibition of the GABA transporter will result in elevation of GABA concentration in the synaptic cleft and hence in stimulation of GABA receptor activity; this could contribute to increased inhibitory synaptic transmission and also to reduced pain sensation. Indeed, experiments with transgenic mice with knockout or overexpressed GABA transporters GAT1 have demonstrated that the GAT1 is correspondingly involved in pain sensation [11]. In these experiments it could also be shown that application of GAT1-selective inhibitors, ethyl nipecotate and NO-711, led to analgesia. Though GAT1 is the dominating neuronal GABA transporter, involvement of nonneuronal transporters cannot be excluded.

GAT1 belongs to a family of secondary active systems (see [12]) that are driven by electrochemica1 gradients for Na^+^ and Cl^−^. The transport of one GABA is coupled to the cotransport of two Na^+^ and one Cl^−^ [13–18]. As a consequence of the stoichiometry, the translocation of GABA across the cell membrane is associated with a current that can be measured under voltage clamp. In the absence of GABA, the transport cycle is not completed, but transient charge movements can be detected; they reflect extracellular Na^+^ binding within the electrical field preceding binding of GABA [19–22]. The GAT1-mediated steady-state current, on the other hand, often reflects only in part the translocation of GABA; another component of GAT1-mediated current represents uncoupled flow of ions through a channel-like mode (see, e.g., [15, 18, 23]).

It was shown previously [24] that acupuncture leads to activation of enkephalinergic neurons and release of endogenous morphines, the endorphins. Activation of opioid receptors has also been shown to regulate pain sensation, and the role of *δ*OR in pain modulation is intriguing [25, 26], Importantly, *δ*OR has been found to be increasingly targeted to the plasma membrane in the spinal cord dorsal horn in inflammation [27]. Loss of synaptic inhibition including GABAergic inhibition in the spinal dorsal horn is considered to contribute significantly to several forms of chronic pain [8, 28], and regulation of GABA transportation has been shown to control pain sensation [11, 29]. Since control and termination of synaptic activity play important roles in physiological and pathophysiological brain functions, regulation of the GABA transporter, which controls the dominating inhibitory transmitter in extracellular space, is a crucial mechanism to regulate neural circuits.

In the present study, potential effects of opiates on GABA transporter were examined, and we found that chronic exposure of rats to morphine reduced GABA uptake into synaptosomes. We have previously shown [9] that the glutamate transporter EAAC1 is downregulated by intermolecular interaction with the Gi-protein-coupled *δ*-opioid receptor of mouse (*δ*OR) [30] and that this inhibitory interaction is counteracted by activation of the *δ*OR [9]. In the work described here, we present evidence that the *δ*OR interferes with functional surface expression of the GABA transporter GAT1 and that *δ*OR activation results in reduced activity of the GAT1.

## 2. Materials and Methods

### 2.1. Experiments on Animals and Synaptosomes

For studying effects of opiates in animal experiments, male Sprague-Dawley rats (200–220 g, Laboratory Animal Center, Chinese Academy of Sciences, Shanghai, China) were used. All experiments were carried out strictly in accordance with the National Institutes of Health Guide for the Care and Use of Laboratory Animals.

Rats were exposed to opioids by s.c. injection of morphine (10 mg/kg) twice per day at 12 h intervals for a period of 10 days as described recently [31, 32]. Control rats were treated similarly, except saline was used throughout. After decapitation of rats, the brains were removed and cooled briefly in chilled balanced salt solution (in mM): 126 NaCl, 5 KCl, 1.25 NaH_2_PO_4_, 10 glucose, 25 NaHCO_3_, 2 CaCl_2_, and 2 MgSO_4_, (pH 7.4). Thereafter, hippocampi were rapidly dissected. Hippocampi express both GAT1 [33] and *δ*OR in GABAergic cells [34]. Subcellular fractions were prepared according to standard methods as described previously [35]. The purified synaptosomes were from P2 fraction of the brain lysate [36].

GABA uptake of synaptosomes was initiated by adding 4 nM [^3^H]-GABA (Amersham Pharmacia Biotech, Buckinghamshire, UK) and 30 *μ*M unlabeled GABA in a final volume of 500 *μ*L KRH (Krebs Ringer's/HEPES) medium [37]. After incubation at 37°C for 5 min, the uptake was terminated by filtration on a GF/C filter (Whatman) under vacuum, and the filter was washed five times with 10 mL of cold KRH medium. Finally, filters containing synaptosomal particles or neuronal lysates were processed for scintillation counting (Beckman Instruments, Torrance, CA). Nonspecific uptake was determined using Na^+^-free media to block GABA transport.

### 2.2. Experiments on Oocytes

*Xenopus* oocytes were obtained as described previously (see, e.g., [38]). Full-grown prophase-arrested oocytes were injected with cRNA for GAT1 or/and *δ*OR of mouse brain or *μ*OR of rat brain, or for the rat *α*2*β* Na^+^, K^+^ pump. cDNA for GAT1 was kindly provided by Dr. Jian Fei (Shanghai, Tongji University). The cells together with noninjected control oocytes were stored at 19°C in oocyte Ringer's solution (ORi (in mM): 90 NaCl, 2 KCl, 2 CaCl_2_, 5 MOPS (adjusted to pH 7.4 with Tris)) containing 70 mg/L gentamicin. The agonist of *δ*OR, [d-Pen^2,5^]-enkephalin (DPDPE), the agonist [d-Ala^2^,N-Me-Phe^4^,Gly^5^-ol]-enkephalin (DAMGO) of *μ*OR, and the general opioid receptor antagonist naloxone (Sigma) were added at the respective concentrations to ORi. Experiments were performed at room temperature (about 22°C) after 3–5 days of incubation.

Membrane currents were recorded under conventional two-electrode voltage clamp (TurboTec, NPI, Tamm, Germany) during rectangular 200 ms voltage-clamp pulses (from −150 to +30 mV in 10 mV increments that were applied from a holding potential of −60 mV (see, e.g., [39]). Voltage dependencies of steady-state and transient membrane currents were analyzed [21]. Steady-state current was determined at the end of the voltage pulses (averaged during the last 20 ms), and transient currents were analyzed from the entire time course during the respective voltage step.

GAT1-mediated currents were calculated as the difference of current in the presence and absence of GABA. Current values determined before and after the application of GABA were averaged to correct for small drifts with time.

To determine maximum transport activity, uptake of  ^3^H-labeled GABA (Amersham, Braunschweig, Germany) was measured at 90 mM external Na^+^ as describe previously [21] using a total concentration of 100 *μ*M GABA. The number of Na^+^, K^+^ pump molecules in the oocyte surface membrane was determined by [^3^H]-ouabain binding; the oocytes were preloaded with Na^+^ by incubating the cells for 40 min in solution that had the following composition (in mM): 110 NaCl, 2.5 sodium citrate, and 5 MOPS (adjusted to pH 7.6). In the loaded oocytes, intracellular activity of Na^+^ was about 80 mM after 40 min of incubation as measured by Na^+^-selective microelectrodes [40]. The number of ouabain binding sites on the oocyte surface was determined in K^+^-free ORi solution containing 2.5 *μ*M [^3^H]ouabain (0.86 TBq/mmol, New England Nuclear) and 2.5 *μ*M of cold ouabain at room temperature [38].

### 2.3. Experiments on Brain Slices

Neonatal Sprague-Dawley rats (10–20 days) were anaesthetized with ether, decapitated and horizontal midbrain slices containing the periaqueductal gray (PAG) were cut (350 *μ*m) in ice-cold (4°C) artificial cerebrospinal fluid (ACSF), and the composition of the ACSF was in mM as follows: NaCl 124, KCl 3.0, NaH_2_PO_4_ 1.25, MgSO_4_·7H_2_O 2.5, NaHCO_3_ 26, glucose-H_2_O 10, and CaCl_2_ 2.0. For recovery the slices were kept at room temperature (25°C) in ACSF equilibrated with 95% O_2_ and 5% CO_2_ for ∼1 hour. The slices were then individually transferred to a chamber and superfused continuously with ACSF (31°C) for electrophysiological experiments.

PAG neurons were visualized using infrared Nomarski optics. Whole-cell recordings were made using patch electrodes (4-5 MΩ); the composition of the electrode filling solution was (in mM) as follows: K-gluconate 95, KCl 30, NaCl 15, MgCl_2_ 2, HEPES 10, EGTA 11, MgATP 2, and NaGTP 0.25 (pH 7.3, 280–285 mOsmol/L). Liquid junction potentials of ∼30 mV were corrected. Series resistance (∼35 MΩ) was compensated automatically and continuously monitored. Whole-cell currents were recorded using EPC10 double amplifier (HEKA Instruments), digitized, filtered (at 2 kHz), and then acquired (sampling at 10 kHz) in PatchMaster (HEKA Instruments). Steady-state currents were determined at the end of 200 ms, rectangular voltage-clamp pulses (from −130 to −30 mV in 10 mV increments, averaged during the last 20 ms) that were applied from a holding potential of −60 mV. GAT1-mediated current was determined as the current component that was inhibited by either 20 *μ*M tiagabine (Biotrend Chemicals AG, Zurich, Switzerland) or 10 *μ*M NNC711 (Tocris Cookson Ltd, Bristol, UK). To reduce background contribution of voltage-gated Na^+^, Ca^2+^, and K^+^ channels, all bath solutions contained 300 nM tetrodotoxin, 10 *μ*M CdCl_2_, and 20 mM tetraethyl ammonium chloride, respectively.

### 2.4. Western Blot

Yolk-free homogenates of oocytes were prepared 3 days after the injection of cRNA as described previously [41] and by passing the oocytes through 200-*μ*L Eppendorf pipette tips in homogenization buffer (in mM: 20 Tris-HCl (pH 7.4), 5 MgCl_2_, 5 NaH_2_PO_4_, 1 EDTA, 100 NaCl, 10 KCl, 1 DTT, and 1 PMSF, and 5 *μ*g/mL of each of leupeptin, pepstatin, and antipain). Twenty-microliter aliquots of the yolk-free homogenates were electrophoresed on SDS-PAGE. Proteins from nonstained gels were electrophoretically transferred on nitrocellulose membrane for western blot. The primary antibody against GAT1 from rabbit (Chemicon Int. AB1570 W) was applied overnight at 4°C. The secondary antibody against GAT1 from rabbit (abcam. ab426) was applied at room temperature for 1 hr. Band intensities were quantified using ImageJ software.

## 3. Results

### 3.1. Exposure of Rats to Morphine Results in Reduced Rate of GABA Uptake

**Figure 1:**
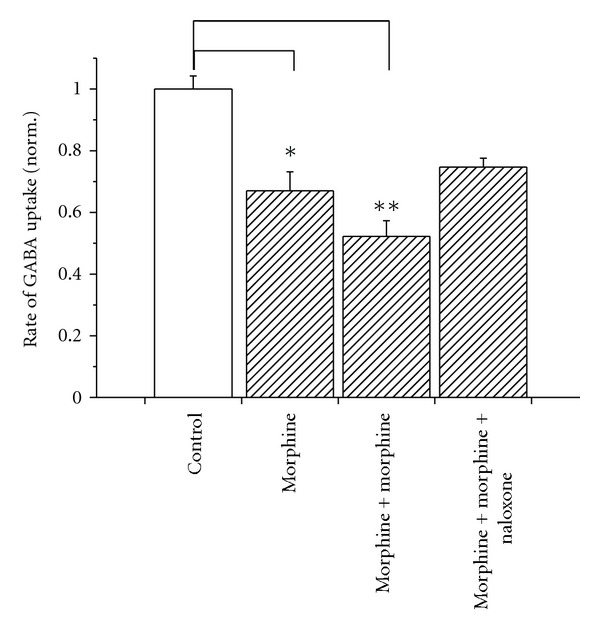
Effect of chronic morphine treatment on GABA uptake activity in rats. Uptake activity was measured in synaptosomes of untreated rats (control) and of rats after 10 days of morphine injection twice a day (hatched bars). Data were normalized to the rate of uptake into synaptosomes of controls. For treated rats, rate of GABA uptake was determined 12 hr after the termination of treatment (morphine), with an additional injection of morphine (s.c. 10 mg/kg) 1 hr before sacrificing (morphine + morphine) or with an additional injection of both morphine and naloxone (i.p. 2 mg/kg) 1 hr before sacrificing (morphine + morphine + naloxone). *P < 0.05, **P < 0.01 compared to data from control animals; *n* = 5 in each group. Error bars represent SEM.

Rate of GABA uptake was determined in synaptosomal preparations of hippocampus of rats that were chronically treated with morphine (Figure 1). Rats were injected twice a day with morphine over a period of 10 days, and GABA uptake into the synaptosomes was significantly reduced 12 h after termination of the morphine treatment compared to untreated controls. Acute injection of morphine (1 h before sacrifice) further decreased the uptake activity. This additional effect of morphine could completely be prevented by the simultaneous use of naloxone, an opioid receptor antagonist, indicating that the acute morphine treatment downregulated GABA transporter activity as a result of activation of opioid receptors.

Among the several canonical opioid receptors, *μ*OR has the highest affinity with morphine [42]; studies with *μ*OR knockout mice also show that *μ*OR is necessary for morphine-induced analgesia and other symptoms [43]. However, at a relatively high concentration, morphine could bind to all three opioid receptors, *μ*OR, *δ*OR, and *κ*OR [44]. In addition, a minor morphine metabolite, morphine-6-glucuronide, showed higher affinity to *δ*OR and lower affinity to *μ*OR, compared to that of morphine in rodents and human [42].

While *μ*OR is constitutively expressed at surface membrane, several studies have shown that the surface expression of *δ*OR is regulated by inflammation, drug exposure, or stimulation [27, 45, 46]. Notably, the physical interactions between *μ*OR and *δ*OR may contribute to long-term changes of morphine-induced analgesia [44, 45]. We reasoned that the changes of either *μ*OR or *δ*OR signaling cascades might be responsible for acute and chronic morphine-induced GABA uptake inhibition. Therefore, we next examined the interaction between GAT1 and *μ*OR or *δ*OR using oocyte as a simplified and well-controlled system.

### 3.2. Effect of Opioid Receptor Coexpression on Rate of GABA Uptake

**Figure 2:**
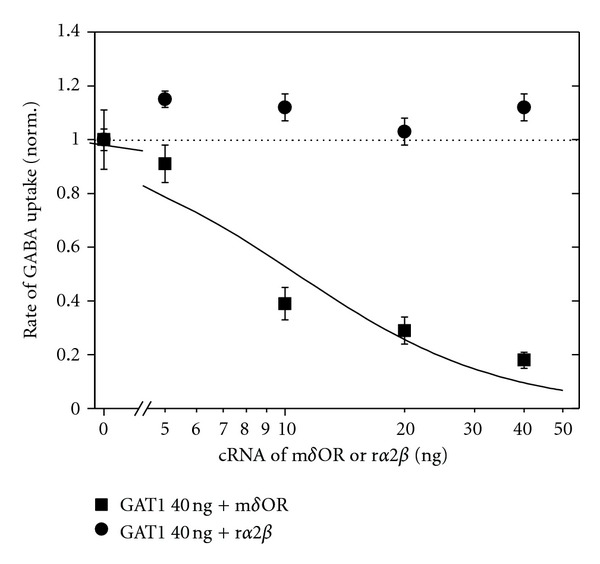
Dependence of GAT1-mediated rate of  [^3^H]GABA uptake on different amounts of coinjected cRNA. For cRNA of *δ*OR (filled squares) or r*α*2*β* pumps (filled circles), the amount of injected cRNA for GAT1 was 40 ng. Data were normalized for each batch of oocytes to the respective value obtained from oocytes not coinjected with cRNA for *δ*OR or r*α*2*β* and are presented as means ± SEM from 2 to 3 batches of oocytes (with 8–10 oocytes per batch). The dependence of rate of GABA uptake on *δ*OR-cRNA was fitted by (1).

**Figure 3:**
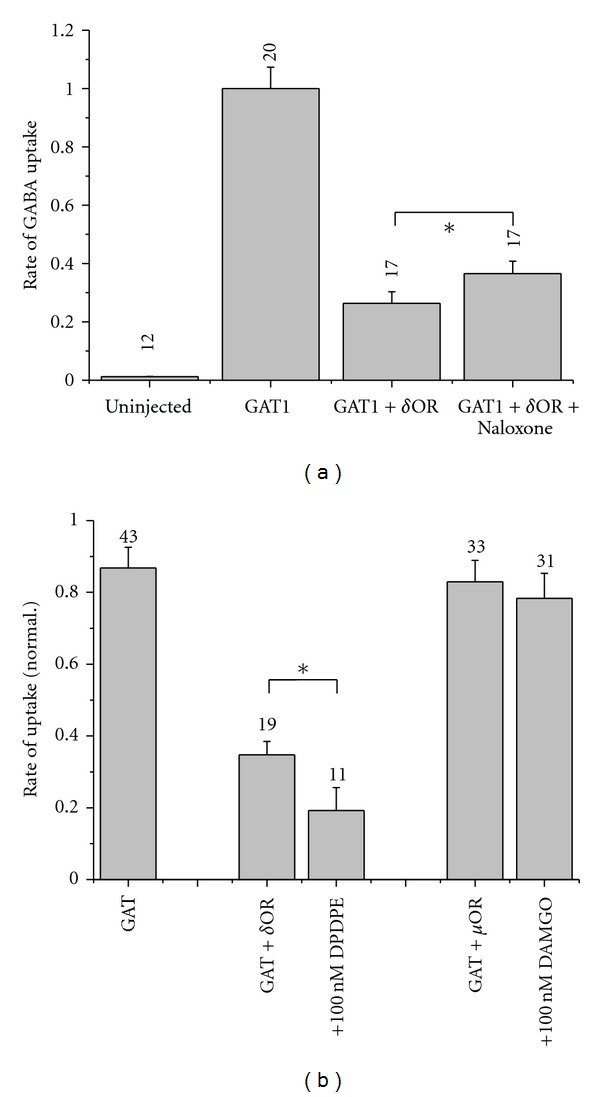
Effect of opioid receptor coexpression on GAT1-mediated rate of GABA uptake. (a) 40 ng of GAT1 cRNA alone or 40 ng of GAT1 and 20 ng of cRNA for *δ*OR were injected into oocytes. Application of 100 nM naloxone gave slight recovery of GAT1 inhibition by the coexpressed *δ*OR. (b) 40 ng of GAT1 cRNA alone or 40 ng of GAT1 and 10 ng of cRNA for *δ*OR or *μ*OR were injected into oocytes. The unspecific rate of uptake was subtracted from the uptake rate of injected oocytes. *δ*OR was activated by application of 100 nM DPDPE, *μ*OR by 100 nM DAMGO. Data were normalized for each batch of oocytes to the respective value obtained from oocytes not coinjected with cRNA for *δ*OR and are presented as means of rates of [^3^H]GABA uptake ± SEM from 2 to 4 batches of oocytes (with 5–10 oocytes per batch), *P < 0.05.

To investigate regulation of GAT1 by opioid receptor, we used the *Xenopus* oocytes with heterologously expressed GAT1 and opioid receptor as a model system. Functional GABA transporters incorporation into the oocyte membrane was verified by detection of Na^+^-dependent uptake of [^3^H]GABA at a saturating GABA concentration of 100 *μ*M [14, 19]. Only oocytes with heterologously expressed GAT1 showed significant [^3^H]GABA uptake (see Figure 3(a)). When GAT1 and the *δ*OR of mouse brain were coexpressed in *Xenopus* oocytes by coinjection of cRNA for GAT1 (40 ng) and different amounts for *δ*OR (0, 5, 10, 20, 40 ng), increasing amounts of the coinjected cRNA of *δ*OR led to decreased rate of GAT1-mediated GABA uptake (Figure 2); the dependency was arbitrarily fitted by

(1) $$\begin{array}{c} \text{Rate}=\frac{K_{1/2}^{n}}{K_{1/2}^{n}+{\left[\text{cRN}{\text{A}}_{\delta \text{OR}}\right]}^{n}} \end{array}$$

with half-maximum inhibition of the rate at *K*_1/2_ = 10.7 ng and *n* = 1.7. Corresponding amounts of cRNA for the rat Na pump, r*α*2*β*, had no significant effect on the GAT1-mediated uptake (Figure 2). To rule out the possibility that expression of *δ*OR in the oocytes may affect translation of other proteins that regulate membrane protein expression, we injected oocytes different amounts of cRNA for *δ*OR together with cRNA for the rat *α*2*β* Na^+^ pump (40 ng). The coexpression of *δ*OR did not affect surface expression of the pump as judged by [^3^H]ouabain binding (not illustrated). For the experiments described in the following, we always choose 10 or 20 ng for coinjection of opioid-receptor cRNA.

The reduced uptake in oocytes expressing both the GAT1 and the *δ*OR compared to those expressing GAT1 alone cannot be attributed to background activation of *δ*OR since treatment with the opioid receptor antagonist naloxone (1 *μ*M) could only slightly reverse the inhibition (Figure 3(a)). Coinjection of 10 ng cRNA for the *μ*OR instead of cRNA for *δ*OR had no significant effect on the rate of GABA uptake (Figure 3(b)). Functional expression of *δ*OR and *μ*OR was confirmed in the absence of GAT1 activity by application of 100 nM of the *δ*OR or *μ*OR agonists, DPDPE or DAMGO, respectively, and by the resulting activation of the endogenous Ca^2+^-activated channels (data not shown, see also [30]); it is worth to mention that the voltage dependencies of the currents induced by DPDPE and DAMGO differ from each other indicating that different signaling pathways are activated.

We further tested the effects of the opioid agonists if 10 ng of cRNA for the respective receptor was coinjected. Figure 3(b) shows that activation of *δ*OR by 100 nM DPDPE further reduced the rate of GABA uptake while activation of *μ*OR by 100 nM DAMGO did not significantly change transport activity. Since the heterologous expression of GAT1 with *δ*OR, but not *μ*OR, exhibited significant effect on GABA uptake, we focused the rest of our study on *δ*OR.

### 3.3. Effect of Opioid Receptor Coexpression on GAT1-Mediated Steady-State Current

**Figure 4:**
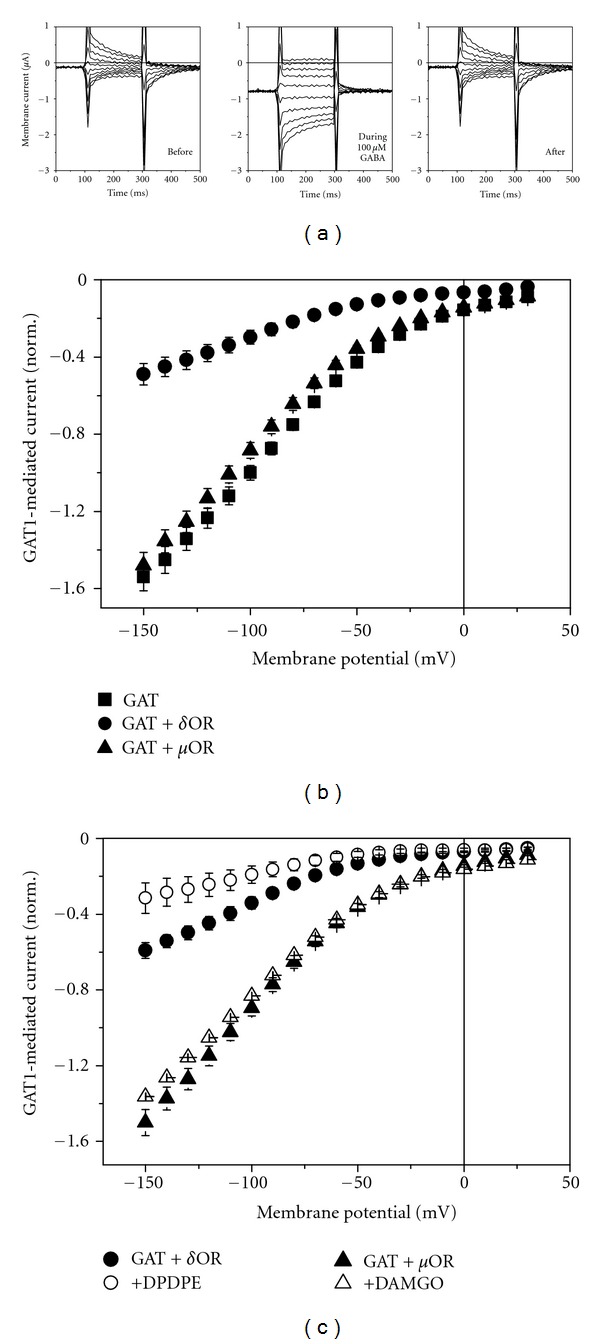
Effects of opioid receptor coexpression on GAT1-mediated current. (a) Current traces in response to rectangular voltage pulses before, during, and after application of 100 *μ*M GABA to oocytes with expressed GAT1. Effects on the voltage dependence of steady-state GAT1-mediated currents in oocytes coexpressed with *δ*OR or *μ*OR (10 ng cRNA coinjected) (b) and of their activation by 100 nM DPDPE or DAMGO, respectively (c). Data in (b) and (c) represent averages ± SEM of at least 6 oocytes.

Dependence of GAT1-mediated current on membrane potential was determined in voltage-clamp experiments as the difference of membrane current in the presence and the absence of 100 *μ*M GABA. Figure 4(a) shows original current traces before, during, and after application of GABA. The steady-state GAT1-mediated current was reduced in the oocytes expressing GAT1 when 10 ng cRNA for *δ*OR were coinjected (Figure 4(b)) to a similar extent as the GABA uptake (compared with Figure 3(b)). As found for the uptake, activation of *δ*OR by application of DPDPE (100 nM) led to further inhibition of GAT1-mediated current by about 50%. Because DPDPE could induce a Ca^2+^-dependent current (see above), the effect of DPDPE on GAT1-mediated current was, therefore, determined in the presence of DPDPE as the difference of current in the presence and absence of GABA.

Similar to the findings with the GABA uptake, coexpression of *μ*OR had no significant effect on the GAT1-mediated steady-state current (Figure 4(b)), and activation of the receptor by 100 nM DAMGO did not significantly influence the current (Figure 4(c)). Also activation of the endogenous acetylcholine (ACh) receptor by 100 *μ*M ACh did not affect the GAT1-mediated current (data not shown).

### 3.4. Effect of Opioid Receptor Coexpression on GAT1-Mediated Transient Current

**Figure 5:**
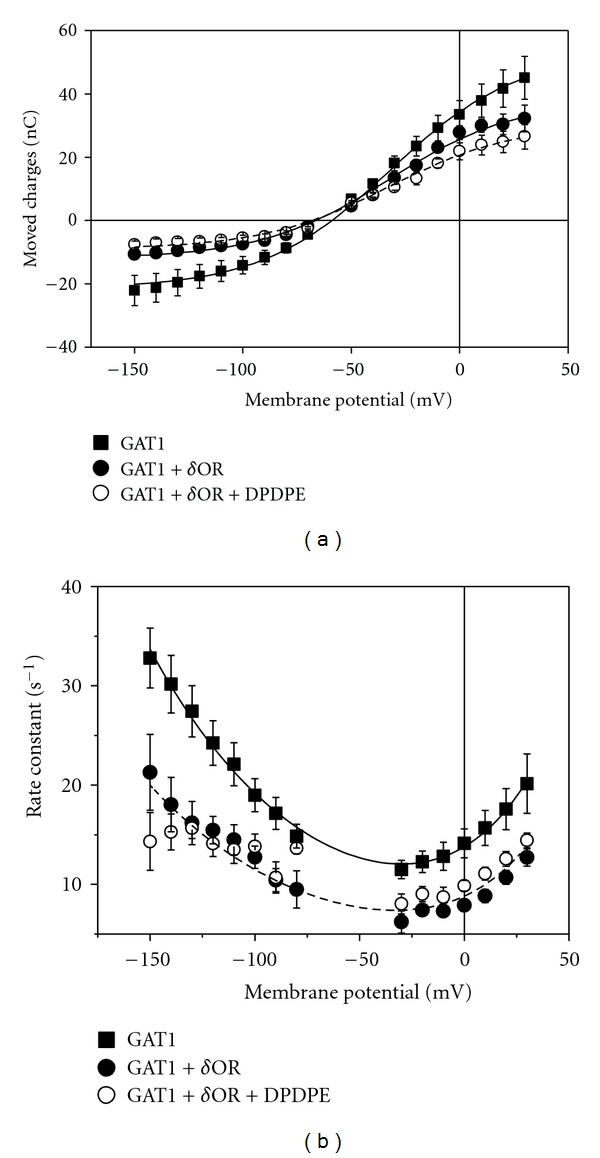
Effects of *δ*OR coexpression and its activation by 100 nM DPDPE on GAT1-mediated transient charge movements. 40 ng of GAT1 cRNA alone or 40 ng of GAT1 and 20 ng of *δ*OR cRNA were injected into oocytes. (a) Voltage dependencies of moved charge in response to rectangular potential jumps were obtained by integration of the respective transient currents and were fitted by (2) (see lines). (b) The rate constants were obtained by fitting an exponential to the transient current signal, and the voltage dependencies were fitted by (3) (see lines). Data represent means ± SEM; *n* = 6-7 oocytes for each group.

Figure 4(a) illustrates, particularly for depolarizing potential steps, that in the absence of GABA a slow transient current was apparent. The amount of the corresponding charge movements *Q* associated with the extracellular Na^+^ binding [19] was determined by integration of the transient current signals remaining after subtracting from the responses in the absence of GABA those in the presence of GABA [20, 21]. The voltage-dependent distribution of the charges *Q*(*E*) is shown in Figure 5(a) and can be described by a Fermi equation:

(2) $$\begin{array}{c} Q\left(E\right)=Q_{-\infty }+\frac{Q_{+\infty }-Q_{-\infty }}{1+e^{-z_{f}(E-E_{1/2})F/RT}}, \end{array}$$

where *F*, *R*, and *T* have their usual meanings, *z*_*f*_ represents the effective valence that is moved during the Na^+^ binding step, and *E*_1/2_ the midpoint potential. Neither *z*_*f*_ nor *E*_1/2_ was affected by coexpression and activation of *δ*OR, only the amount of moved charges became reduced (see Table 1). From the ratio of total charge *Q*_max⁡_ = *Q*_+*∞*_ − *Q*_−*∞*_ and the effective valence *z*_*f*_, the number of functionally expressed transporters *N* can be calculated, and the values are listed in Table 1. The coexpression of *δ*OR obviously led to a reduction in the number of functioning transporters by about 30% which can only partially account for the reduction of GABA uptake (about 70%, see Figure 3(b)) or of GAT1-mediated current (about 75%, see Figures 4(b) and 4(c)). Also the further inhibition of the current by DPDPE application (about 50%, see Figure 4(c)) can only partially be attributed to a reduction in *N* which amounts to only 25%.

Analysis of the kinetics of the transient currents yields rate constants *k* (Figure 5(b)) of the signals that were obtained by fitting *I* = *I*_max⁡_ *e* ^−*kt*^ to the transient signal. The voltage dependencies of the *k* values were fitted by

(3) $$\begin{array}{c} {k(V)=k_{1}(V)+k_{2}(V)=k_{1}^{*}e^{-a_{1}V}+k_{2}^{*}e}^{+a_{2}V}, \end{array}$$

where *k*_1_ and *k*_2_ represent the forward and backward rate constants, respectively, of a step associated with the extracellular Na^+^ binding (fit parameters, see Table 1). All datasets could be fitted by the same voltage dependency; the slowed kinetics on coexpression of *δ*OR was dominated by the reduced forward rate constant that could account for reduced rate of GABA transport. Activation of *δ*OR by DPDPE did not affect the kinetics. Neither the GAT1-mediated steady-state currents nor the transient charge movements were significantly affected when the r*α*2*β* Na^+^ pump was coexpressed with the GAT1.

**Figure 6:**
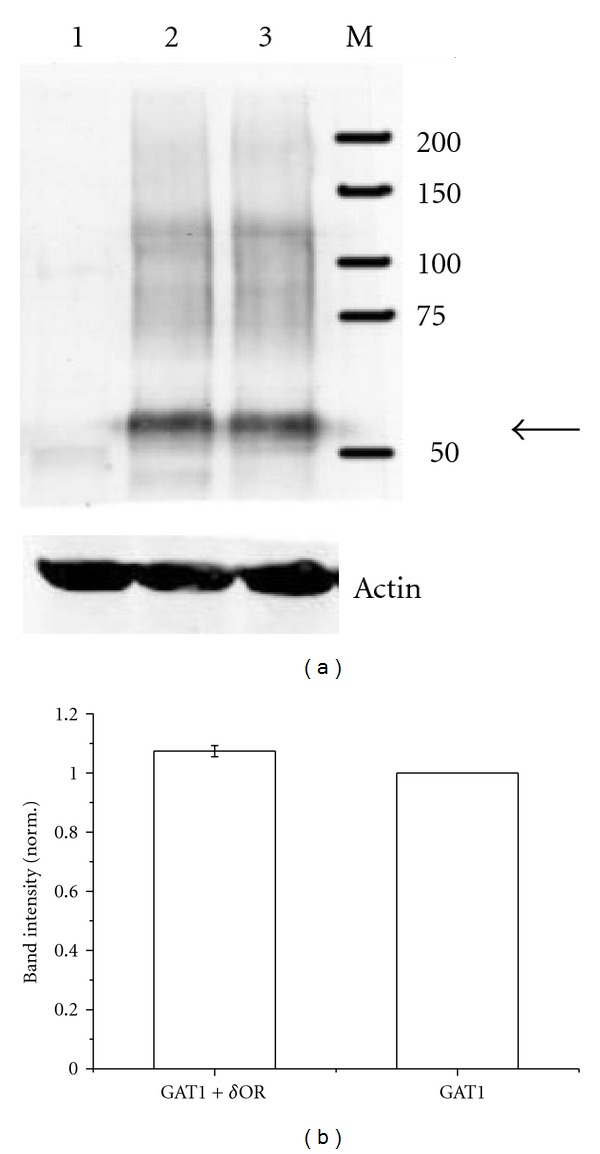
GAT1 expression in *Xenopus* oocyte membrane fractions. (a) Lane 1: noninjected oocytes, lane 2: oocytes injected with 40 ng of GAT1 cRNA and 20 ng of *δ*OR, and lane 3: oocytes injected with 40 ng of GAT1 cRNA alone. The bands at about 60 kDa (see arrow) represent GAT1 monomers. (b) GAT1 monomer band intensities averaged from 5 batches of oocytes and normalized to the respective batch injected with cRNA for GAT1 only.

Western blot analysis shows that coexpression of *δ*OR does not significantly affect the band intensity for GAT1 (Figure 6(a)). If any there may be a slight increase in band intensity with coexpressed *δ*OR of 7.4 ± 1.9% (Figure 6(b)). Same loading of the lanes was confirmed by same band intensities for actin.

### 3.5. Activation of *δ*-Opioid Receptor Inhibits GAT1-Mediated Current in PAG Neurons

The above data have demonstrated that *δ*OR interferes with GAT1 activity; in particular, activation of the opioid receptor inhibits GAT1 function. Since these effects might be a result of overexpression in the oocyte model system, we investigated the effect of *δ*OR activation in brain slices of rat PAG.

Steady-state currents were determined during superfusion of the brain slice with different solutions; in a typical experiment sequence was

(4) $$\begin{array}{c} {\text{S}}_{\text{GABA}}\to {\text{S}}_{\text{GABA}+\text{inhibitor}}\to {\text{S}}_{\text{GABA}} \end{array}$$

with S_GABA_ representing solution with GABA and S_GABA+inhibitor_ solution with additional GAT1-specific inhibitor tiagabine or NNC711. To determine GAT1-mediated current, the currents in S_GABA_ before and after application of the inhibitor were averaged, and the current in the presence of the inhibitor was subtracted to obtain the current component sensitive to the specific inhibitor. Thereafter, solutions in the presence of the *δ*OR against DPDPE were applied:

(5) $$\begin{array}{c} {\text{S}}_{\text{GABA}}\to {\text{S}}_{\text{GABA}+\text{inhibitor}}. \end{array}$$

**Figure 7:**
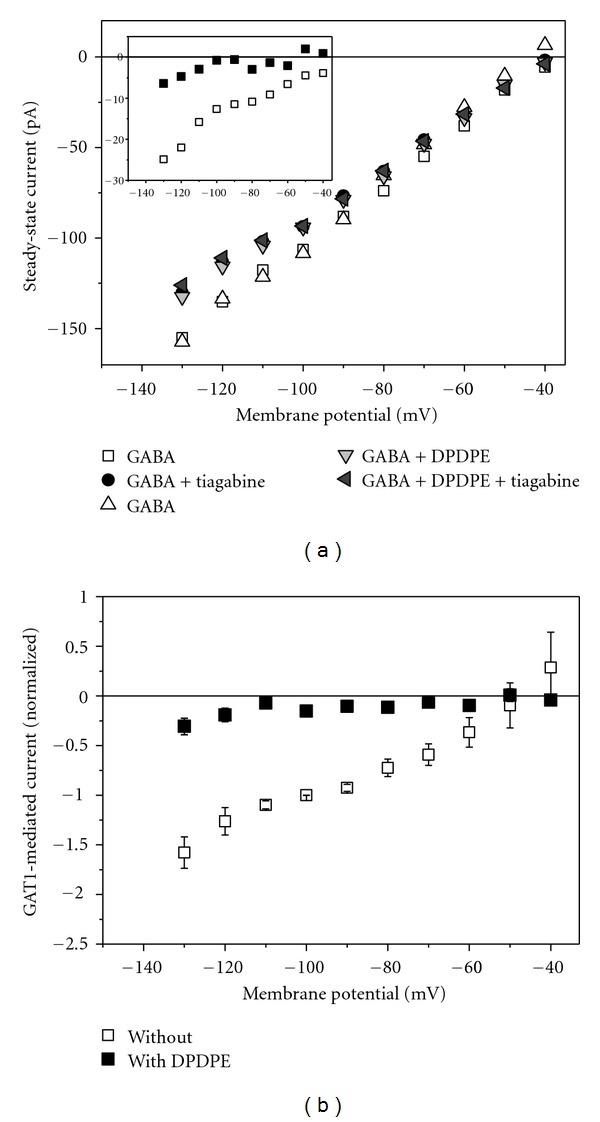
Effect of *δ*OR activation on GAT1-mediated current in rat brain slices from PAG area. Whole-cell patch-clamp recordings were performed on PAG neurons superfusing with different solutions. (a) Steady-state current voltage dependencies of a single experiment with solution sequence as given in the text. The inset shows the GAT1-mediated current determined as tiagabine-sensitive steady-state current. Open squares represent current in the absence and filled squares in the presence of 100 nM DPDPE. (b) GAT1-mediated current from 5 experiments. Data were normalized to the current at −100 mV in the absence of DPDPE and represent averages (±SEM). The value of “one” corresponds to 41.4 ± 20.0 pA.

Correspondingly, the GAT1-mediated current was determined as the difference of current in the absence and presence of the GAT1 inhibitor. Figure 7(a) shows an example. For the GAT1-mediated current averaged data are presented in Figure 7(b).

The result revealed strong inhibition of GAT1-mediated current in response to application of the *δ*OR-specific agonist DPDPE (Figure 7). The nearly complete inhibition of total GAT1-mediated current indicates that in the brain slices all transport modes of GAT1 were blocked. Hence, these data on PAG slices are consistent with the DPDPE-induced reduction of GAT1-mediated GABA uptake and current observed in oocytes.

## 4. Discussion

The discovery that the opioid receptor antagonist naloxone counteracts acupuncture-induced analgesia [47, 48] led to the suggestion of the involvement of endorphins. Administration of opiates to rats resulted in reduced GABA uptake in brain as determined from measurements in synaptosomes (Figure 1). The GABAergic system also plays a critical role in pain sensation, and in particular inhibition of GAT in mice has antinociceptive effects and transgenic GAT1-overexpressing mice are hyperanalgesic [11]. Since the GABA concentration is controlled to a large extent by the GABA uptake transporter, we investigated effects of opioid receptor expression on GAT1 function. To avoid interference with other GABAergic components, we used the *Xenopus* oocytes as an expression system. This was also useful since the oocytes do not express endogenous functional Na^+^ channels that have been demonstrated to be also regulated by *δ*OR [49].

### 4.1. Coexpression of *δ*OR Reduces Transport Mediated by GAT1

Coexpression of the *δ*OR with GAT1 from mouse brain led to downregulation of GABA uptake in the oocytes with half maximum inhibition at about 10 ng of cRNA of *δ*OR (Figure 2); this means at a cRNA_*δ*OR_/cRNA_GAT1_ ratio of 0.25. The GAT1-mediated current was also reduced (Figure 4(b)). The effects of *δ*OR coexpression on GABA transport cannot be attributed to competition by the coinjected cRNAs. Neither the translation machinery nor the targeting to the surface membrane was a limiting factor. This became apparent from the observation that GABA uptake was hardly affected by coexpression of another membrane protein (Na^+^ pump), and Na^+^-pump expression was not affected by *δ*OR expression (Figure 2). The reduced transport without activation of *δ*OR cannot be attributed to a background activity of the receptor since application of the opioid-receptor antagonist naloxone did not have a compensating counteractive effect. Only a slightly stimulated uptake could be detected in the presence of 100 nM naloxone (Figure 3(a)). *δ*OR seems to directly interfere with the GAT1 leading to the reduced GABA uptake. This effect was specific for *δ*OR since coexpression of *μ*OR did affect neither GAT1-mediated GABA uptake (Figure 3(b)) nor current (Figure 4(b)); also transport activities of EAAC1 and the Na, K pump were not affected [9, 50, 51]. Interaction of membrane receptors with transport systems without activation of the receptor was also reported previously. The glutamate transporter EAAC1 as well as the Na, K pump showed reduced transporter activity with coexpressed *δ*OR [9, 50, 51], and for the dopamine transporter DAT reduced [52] as well as increased [53] uptake was reported for coexpression with G-protein receptors. This effect was specific for *δ*OR since coexpression of *μ*OR did affect neither GAT1-mediated GABA uptake (Figure 3(b)) nor current (Figure 4(b)); also transport activities of EAAC1 and the Na, K pump were not affected [9, 50, 51].

The specificity of the effect of coexpression of *δ*OR on the GABA transporter becomes also apparent from the observation that targeting of the rat *α*2*β* Na^+^, K^+^ pumps to the surface membrane of the oocyte was not affected by coexpression of *δ*OR (Figure 2). In addition, coexpression of the rat Na^+^, K^+^ pump neither reduced GABA uptake nor GAT1-mediated current, and the number of transporters was not significantly affected either (data not shown).

Western blot analysis also demonstrated that coexpression of *δ*OR did not affect the amount of GAT1 expressed in the oocytes and targeted to the membrane. Nevertheless, the number of functioning transporters calculated from the *Q*_max⁡_ value became reduced by about 30% (see Table 1) on coexpression of *δ*OR. “Functioning transporter” means that at least Na^+^ can still bind giving rise to the transient current signal, but it does not necessarily mean that GABA can be transported. The inhibition of transport, therefore, might be attributed to a reduced number of GABA-translocating GAT1 molecules in the membrane and to a reduced rate of transport. The reduced apparent affinity for extracellular Na^+^ binding (reduced rate of binding and increased rate of unbinding (Figure 5(b)) supports the view of a contribution of a reduced turnover rate of the transport cycle.

### 4.2. Activation of *δ*OR Reduces Transport Mediated by GAT1

As a supraspinal locus for opioid analgesia PAG exhibits high abundance of *δ*OR (see, e.g., [54, 55]) and also expression of GAT1 has been demonstrated [56]. In *Xenopus* oocytes with coexpressed *δ*OR and GAT1 activation of *δ*OR by DPDPE led to further reduction of GAT1-mediated current (Figure 4(c)) compatible with the finding of reduced GABA uptake activity (Figure 3(b)). Compared to nonstimulated *δ*OR, the inhibition of current amounts to 41% and of uptake to 45%.

Redistribution of transporters between cytoplasmic membranes and the surface membrane resulting in altered transport had been attributed to activity of protein kinase C (PKC) [57]. Opioid receptor activation with activation of PKC had indeed been observed previously [58, 59]. Hence we may speculate that the inhibition of GAT1 activity by *δ*OR activation might be regulated by PKC; an altered GAT1-*δ*OR interaction might also be considered as had been suggested for the stimulation of glutamate uptake on activation of *δ*OR [9]. This of course needs further investigations.

Altered GABA transport as a consequence of activation of G-protein-coupled receptor has been discussed on the basis of indirect effects through altered Na^+^ gradient [60]. Such a mechanism can be excluded for the oocytes since intracellular Na^+^ activity hardly changes during an experiment, and more direct effects (see, e.g., [61]) have to be considered including the action of protein kinases and phosphatases. Stimulation of PKC alters GABA uptake by redistribution of the transporters between intracellular compartments and the surface membrane [57, 62], but also reduced activity of the GABA transporter has been observed (Eckstein-Ludwig and Schwarz (unpublished)). Mechanisms have been discussed that protein-protein interaction like interaction with syntaxin A or other adaptor proteins modulates the GABA transporter [63] that may be modulated by PKC [64]. Also other transport proteins, channels, G-protein-coupled receptors, and cytoplasmic proteins [65–68] seem to be affected by protein-protein interactions. 

The inhibition of GAT1-mediated current cannot be attributed to an overexpression of GAT1 and/or *δ*OR in the model system “*Xenopus* oocyte.” This we demonstrated by patch-clamp experiments on PAG brain slices (Figure 7); application of the *δ*OR-specific agonist DPDPE resulted in nearly complete inhibition of tiagabine-sensitive current that can be considered to be mediated by GAT1. In *Xenopus* oocyte activation of *δ*OR did not completely inhibit the GAT1-mediated current as well as uptake. This may be due to the ratio of injected cRNA for GAT1 and *δ*OR; we found (not shown) that the degree of uptake inhibition by *δ*OR activation increases with lower amounts of injected cRNA for *δ*OR.

The signaling pathway in the oocytes definitively differs from that in the brain and might be the reason for the differently pronounced effects on the GAT1 transport modes. Our results suggest that stimulation of *δ*OR can modulate the GAT1 activity. Interestingly, while *δ*OR activation leads to inhibition of GAT1-mediated current, the glutamate transporter EAAC1 becomes stimulated [9]. In addition to this modulation of synaptic transmission, modulation of neurotransmitter release by stimulation of opioid receptors has been reported [69, 70].

In the presented work we have demonstrated that GAT1-mediated transport can be regulated by the G-protein-coupled *δ*-opioid receptor in two ways: (1) by modulation of the number of functional transporters in the membrane and (2) by modulation of transport activity. These modulations are dominated by the presence of *δ*OR and by receptor activation suggesting that direct transporter-receptor interaction plays the dominating role. Chronic morphine treatment is known to modulate the surface expression of *δ*OR. For example, it blocks agonist-induced *δ*OR internalization [71]. The dynamics of *δ*OR surface expression will thus regulate GABA transporter activity. Since reduced GABA reuptake will lead to elevated GABA concentration in the synaptic cleft, this mechanism may account for the increased GABAergic activity found in chronic opiate-treated rats and its role in pain sensation.

## 5. Conclusion

It has been show previously that Na^+^ channel inhibition by *δ*OR activation may contribute to attenuation of disrupture of ionic homeostasis present under hypoxic/ischemic conditions [72]. Activation of *δ*OR can stimulate the neurotransmitter transporters EAAC1 [9] and inhibit the neurotransmitter transporter GAT1 (this work). Stimulation of EAAC1 will lead to reduced concentration of the excitatory neurotransmitter glutamate in the synaptic cleft and inhibition of GAT1 to elevated concentration of the inhibitory transmitter GABA. We like to suggest that the acupuncture-induced elevation of endorphins [24] can contribute to analgesia by regulation of the dominating excitatory and inhibitory neurotransmitter transporters by activation of *δ*OR.

## Figures and Tables

**Table 1:** Parameters of (1) fitted to the data shown in Figure 5(a).

	*Q*_max⁡_ (nC)	*E*_1/2_ (mV)	*z*_*f*_	*Q*_max⁡_/*z*_*f*_ (*N* per oocyte)	*k*_1_* (s^−1^)	*a*_1_ (mV^−1^)	*k*_2_* (s^−1^)	*a*_2_ (mV^−1^)
GAT1	73.3	−32.3	1.2	6.1 × 10^11^	6.8	85.6	7.8	39.5
GAT1 + *δ*OR	49.4			4.1 × 10^11^	3.3		5.4	
GAT1 + *δ*OR (1 *μ*M DPDPE)	39.7			3.3 × 10^11^				

## Abbreviations

ACSF: Artificial cerebrospinal fluid
DAMGO: [d-Ala^2^,N-Me-Phe^4^,Gly^5^-ol]-enkephalin
*δ*OR: *δ*-Opioid receptor
*μ*OR: *μ*-Opioid receptor
DPDPE: [d-Pen^2,5^]-Enkephalin
EAAC1: Excitatory amino acid carrier (isoform 1)
GABA: *γ*-Amino butyric acid
GAT1: GABA transporter (isoform 1)
MOPS: 3-(N-morpholino)-propanesulphonic acid
ORi: Oocyte Ringer's solution
PAG: Periaqueductal gray
PKC: Protein kinase C
r*α*2*β* pump: Na^+^ pump of *α*2 and *β* subunit of rat.

## References

[1] Rothstein JD, Martin L, Levey AI (1994). Localization of neuronal and glial glutamate transporters. *Neuron*.

[2] He Y, Janssen WG, Rothstein JD, Morrison JH (2000). Differential synaptic localization of the glutamate transporter EAAC1 and glutamate receptor subunit GluR2 in the rat hippocampus. *The Journal of Comparative Neurology*.

[3] Levenson J, Weeber E, Selcher JC, Kategayal LS, Sweatt DJ, Eskin A (2002). Long-term potentiation and contextual fear conditioning increase neuronal glutamate uptake. *Nature Neuroscience*.

[4] Borden LA, Caplan MJ (1996). GABA transporter heterogeneity: pharmacology and cellular localization. *Neurochemistry International*.

[5] Malan TP, Mata HP, Porreca F (2002). Spinal GABAA and GABAB receptor pharmacology in a rat model of neuropathic pain. *Anesthesiology*.

[6] Zhang YQ, Ji GC, Wu GC, Zhao ZQ (2002). Excitatory amino acid receptor antagonists and electroacupuncture synergetically inhibit carrageenan-induced behavioral hyperalgesia and spinal fos expression in rats. *Pain*.

[7] Zhang J, Gibney GT, Zhao P, Xia Y (2002). Neuroprotective role of *δ*-opioid receptors in cortical neurons. *American Journal of Physiology*.

[8] Knabl J, Witschi R, Hösl K (2008). Reversal of pathological pain through specific spinal GABAA receptor subtypes. *Nature*.

[9] Xia P, Pei G, Schwarz W (2006). Regulation of the glutamate transporter EAAC1 by expression and activation of *δ*-opioid receptor. *European Journal of Neuroscience*.

[10] Schwarz W, Gu QB, Xia Y, Ding GH, Wu G-C (2012). Cellular mechanisms in acupuncture points and affected sites. *Current Research in Acupuncture*.

[11] Hu JH, Yang N, Ma YH (2003). Hyperalgesic effects of *γ*-aminobutyric acid transporter I in mice. *Journal of Neuroscience Research*.

[12] Schloss P, Mayser W, Betz H (1992). Neurotransmitter transporters. A novel family of integral plasma membrane proteins. *FEBS Letters*.

[13] Keynan S, Kanner BI (1988). *γ*-Aminobutyric acid transport in reconstituted preparations from rat brain: coupled sodium and chloride fluxes. *Biochemistry*.

[14] Kavanaugh MP, Arriza JL, North RA, Amara SG (1992). Electrogenic uptake of *γ*-aminobutyric acid by a cloned transporter expressed in Xenopus oocytes. *Journal of Biological Chemistry*.

[15] Risso S, DeFelice LJ, Blakely RD (1996). Sodium-dependent GABA-induced currents in GAT1-transfected HeLa cells. *Journal of Physiology*.

[16] Hilgemann DW, Lu CC (1999). GAT1 (GABA:Na^+^:Cl^−^) cotransport function: database reconstruction with an alternating access model. *Journal of General Physiology*.

[17] Loo DDF, Eskandari S, Boorer KJ, Sarkar HK, Wright EM (2000). Role of Cl^−^ in electrogenic NA^+^-coupled cotransporters GAT1 and SGLT1. *Journal of Biological Chemistry*.

[18] Krause S, Schwarz W (2005). Identification and selective inhibition of the channel mode of the neuronal GABA transporter 1. *Molecular Pharmacology*.

[19] Mager S, Naeve J, Quick M, Labarca C, Davidson N, Lester HA (1993). Steady states, charge movements, and rates for a cloned GABA transporter expressed in *Xenopus oocytes*. *Neuron*.

[20] Mager S, Kleinberger-Doron N, Keshet GI, Davidson N, Kanner BI, Lester HA (1996). Ion binding and permeation at the GABA transporter GAT1. *Journal of Neuroscience*.

[21] Liu Y, Eckstein-Ludwig U, Fei J, Schwarz W (1998). Effect of mutation of glycosylation sites on the Na^+^ dependence of steady-state and transient currents generated by the neuronal GABA transporter. *Biochimica et Biophysica Acta*.

[22] Bicho A, Grewer C (2005). Rapid substrate-induced charge movements of the GABA transporter GAT1. *Biophysical Journal*.

[23] Eckstein-Ludwig U, Fueta Y, Fei J, Schwarz W, Suketa Y, Carafoli E, Lazdunski M, Mikoshiba K, Okada Y, Wright EM (2000). The neuronal GABA transporter GAT1 as a target for action of antiepileptic drugs. *Control and Diseases of Sodium Transport Proteins and Channels*.

[24] Han JS (2004). Acupuncture and endorphins. *Neuroscience Letters*.

[25] Heinricher MM, Fields H, Chang KJ, Porreca F, Woods J (2003). Molecular and effect of delta opioid compounds. *The Delta Receptor*.

[26] Fields H (2004). State-dependent opioid control of pain. *Nature Reviews Neuroscience*.

[27] Cahill CM, Morinville A, Hoffert C, O’Donnell D, Beaudet A (2003). Up-regulation and trafficking of *δ* opioid receptor in a model of chronic inflammation: implications for pain control. *Pain*.

[28] Moore KA, Kohno T, Karchewski LA, Scholz J, Baba H, Woolf CJ (2002). Partial peripheral nerve injury promotes a selective loss of GABAergic inhibition in the superficial dorsal horn of the spinal cord. *Journal of Neuroscience*.

[29] Jasmin L, Wu MV, Ohara PT (2004). GABA puts a stop to pain. *CNS and Neurological Disorders*.

[30] Miyamae T, Fukushima N, Misu Y, Ueda H (1993). *δ* Opioid receptor mediates phospholipase C activation via G_i_ in *Xenopus* oocytes. *FEBS Letters*.

[31] Trujillo KA, Akil H (1991). Inhibition of morphine tolerance and dependence by the NMDA receptor antagonist MK-801. *Science*.

[32] Pu L, Bao GB, Xu NJ, Ma L, Pei G (2002). Hippocampal long-term potentiation is reduced by chronic opiate treatment and can be restored by re-exposure to opiates. *Journal of Neuroscience*.

[33] Gong N, Li Y, Cai GQ (2009). GABA transporter-1 activity modulates hippocampal theta oscillation and theta burst stimulation-induced long-term potentiation. *Journal of Neuroscience*.

[34] Erbs E, Faget L, Scherrer G (2012). Distribution of delta opioid receptor-expressing neurons in the mouse hippocampus. *Neuroscience*.

[35] Ortiz J, Harris HW, Guitart X, Terwilliger RZ, Haycock JW, Nestler EJ (1995). Extracellular signal-regulated protein kinases (ERKs) and ERK kinase (MEK) in brain: regional distribution and regulation by chronic morphine. *Journal of Neuroscience*.

[36] Xu NJ, Bao L, Fan HP (2003). Morphine withdrawal increases glutamate uptake and surface expression of glutamate transporter GLT1 at hippocampal synapses. *Journal of Neuroscience*.

[37] Ullensvang K, Lehre KP, Storm-Mathisen J, Danbolt NC (1997). Differential developmental expression of the two rat brain glutamate transporter proteins GLAST and GLT. *European Journal of Neuroscience*.

[38] Schmalzing G, Gloor S, Omay H, Kroner S, Appelhans H, Schwarz W (1991). Up-regulation of sodium pump activity in *Xenopus laevis* oocytes by expression of heterologous *β*1 subunits of the sodium pump. *Biochemical Journal*.

[39] Lafaire AV, Schwarz W (1986). Voltage dependence of the rheogenic Na^+^/K^+^ ATPase in the membrane of oocytes of *Xenopus laevis*. *Journal of Membrane Biology*.

[40] Vasilets LA, Ohta T, Noguchi S, Kawamura M, Schwarz W (1993). Voltage-dependent inhibition of the sodium pump by external sodium: species differences and possible role of the N-terminus of the *α*-subunit. *European Biophysics Journal*.

[41] Zhu Y, Vasilets LA, Fei J, Guo L, Schwarz W (2004). Different functional roles of arginine residues 39 and 61 and tyrosine residue 98 in transport and channel mode of the glutamate transporter EAAC1. *Biochimica et Biophysica Acta*.

[42] Oguri K, Yamada-Mori I, Shigezane J (1987). Enhanced binding of morphine and nalorphine to opioid delta receptor by glucuronate and sulfate conjugations at the 6-position. *Life Sciences*.

[43] Matthes HWD, Maldonado R, Simonin F (1996). Loss of morphine-induced analgesia, reward effect and withdrawal symptoms in mice lacking the *μ*-opioid-receptor gene. *Nature*.

[44] Constantino CM, Gomes I, Stockton SD, Lim MP, Devi LA (2012). Opioid receptor heteromers in analgesia. *Expert Reviews in Molecular Medicine*.

[45] Zhang X, Bao L, Guan JS (2006). Role of delivery and trafficking of *δ*-opioid peptide receptors in opioid analgesia and tolerance. *Trends in Pharmacological Sciences*.

[46] Commons KG (2003). Translocation of presynaptic delta opioid receptors in the ventrolateral periaqueductal gray after swim stress. *Journal of Comparative Neurology*.

[47] Pomeranz B, Chiu D (1976). Naloxone blockade of acupuncture analgesia: endorphin implicated. *Life Sciences*.

[48] Mayer DJ, Price DD, Rafii A (1977). Antagonism of acupuncture analgesia in man by the narcotic antagonist naloxone. *Brain Research*.

[49] Kang X, Chao D, Gu Q (2009). *δ*-Opioid receptors protect from anoxic disruption of Na^+^ homeostasis via Na^+^ channel regulation. *Cellular and Molecular Life Sciences*.

[50] Deng H, Yang Z, Li Y (2009). Interactions of Na^+^,K^+^-ATPase and co-expressed *δ*-opioid receptor. *Neuroscience Research*.

[51] Yang ZJ, Bao GB, Deng HP (2008). Interaction of *δ*-opioid receptor with membrane transporters: possible mechanisms in pain suppression by acupuncture. *Journal of Acupuncture and Tuina Science*.

[52] Marazziti D, Mandillo S, Di Pietro C, Golini E, Matteoni R, Tocchini-Valentini GP (2007). GPR37 associates with the dopamine transporter to modulate dopamine uptake and behavioral responses to dopaminergic drugs. *Proceedings of the National Academy of Sciences of the United States of America*.

[53] Lee FJS, Pei L, Moszczynska A, Vukusic B, Fletcher PJ, Liu F (2007). Dopamine transporter cell surface localization facilitated by a direct interaction with the dopamine D2 receptor. *EMBO Journal*.

[54] Bie B, Pan ZZ (2007). Trafficking of central opioid receptors and descending pain inhibition. *Molecular Pain*.

[55] Mansour A, Fox CA, Akil H, Watson SJ (1995). Opioid-receptor mRNA expression in the rat CNS: anatomical and functional implications. *Trends in Neurosciences*.

[56] Barbaresi P, Gazzanelli G, Malatesta M (1998). GABA transporter-1 (GAT-1) immunoreactivity in the cat periaqueductal gray matter. *Neuroscience Letters*.

[57] Quick MW, Corey JL, Davidson N, Lester HA (1997). Second messengers, trafficking-related proteins, and amino acid residues that contribute to the functional regulation of the rat brain GABA transporter GAT1. *Journal of Neuroscience*.

[58] Lou LG, Ma L, Pei G (1997). Nociceptin/Orphanin FQ activates protein kinase C, and this effect is mediated through phospholipase C/Ca^2+^ pathway. *Biochemical and Biophysical Research Communications*.

[59] Lou LG, Pei G (1997). Modulation of protein kinase C and cAMP-dependent protein kinase by *δ*-opioid. *Biochemical and Biophysical Research Communications*.

[60] Schoffelmeer ANM, Vanderschuren LJMJ, De Vries TJ, Hogenboom F, Wardeh G, Mulder AH (2000). Synergistically interacting dopamine D1 and NMDA receptors mediate nonvesicular transporter-dependent GABA release from rat striatal medium spiny neurons. *Journal of Neuroscience*.

[61] Beckman ML, Quick MW (1998). Neurotransmitter transporters: regulators of function and functional regulation. *Journal of Membrane Biology*.

[62] Corey JL, Davidson N, Lester HA, Brecha N, Quick MW (1994). Protein kinase C modulates the activity of a cloned *γ*-aminobutyric acid transporter expressed in Xenopus oocytes via regulated subcellular redistribution of the transporter. *Journal of Biological Chemistry*.

[63] Deken SL, Beckman ML, Boos L, Quick MW (2000). Transport rates of GABA transporters: regulation by the N-terminal domain and syntaxin 1A. *Nature Neuroscience*.

[64] Beckman ML, Bernstein EM, Quick MW (1998). Protein kinase C regulates the interaction between a GABA transporter and syntaxin 1A. *Journal of Neuroscience*.

[65] Ogimoto G, Yudowski GA, Barker CJ (2000). G protein-coupled receptors regulate Na^+^,K^+^-ATPase activity and endocytosis by modulating the recruitment of adaptor protein 2 and clathrin. *Proceedings of the National Academy of Sciences of the United States of America*.

[66] Shenoy SK, Lefkowitz RJ (2003). Multifaceted roles of *β*-arrestins in the regulation of seven-membrane-spanning receptor trafficking and signalling. *Biochemical Journal*.

[67] El Far O, Betz H (2002). G-protein-coupled receptors for neurotransmitter amino acids: C-terminal tails, crowded signalosomes. *Biochemical Journal*.

[68] Zhu Y, Fei J, Schwarz W (2005). Expression and transport function of the glutamate transporter EAAC1 in Xenopus oocytes is regulated by syntaxin 1A. *Journal of Neuroscience Research*.

[69] Illes P (1989). Modulation of transmitter and hormone release by multiple neuronal opioid receptors.. *Reviews of Physiology Biochemistry and Pharmacology*.

[70] Heijna MH, Hogenboom F, Mulder AH, Schoffelmeer ANM (1992). Opioid receptor-mediated inhibition of ^3^H-dopamine and ^14^C-acetylcholine release from rat nucleus accumbens slices. A study on the possible involvement of K^+^ channels and adenylate cyclase. *Naunyn-Schmiedeberg’s Archives of Pharmacology*.

[71] Eisinger DA, Ammer H, Schulz R (2002). Chronic morphine treatment inhibits opioid receptor desensitization and internalization. *Journal of Neuroscience*.

[72] Chao D, Xia Y (2010). Ionic storm in hypoxic/ischemic stress: can opioid receptors subside it?. *Progress in Neurobiology*.

